# The Follow-Up of Chinese Patients in cblC Type Methylmalonic Acidemia Identified Through Expanded Newborn Screening

**DOI:** 10.3389/fgene.2022.805599

**Published:** 2022-02-15

**Authors:** Shiying Ling, Shengnan Wu, Ruixue Shuai, Yue Yu, Wenjuan Qiu, Haiyan Wei, Chiju Yang, Peng Xu, Hui Zou, Jizhen Feng, Tingting Niu, Haili Hu, Huiwen Zhang, Lili Liang, Deyun Lu, Zhuwen Gong, Xia Zhan, Wenjun Ji, Xuefan Gu, Lianshu Han

**Affiliations:** ^1^ Department of Pediatric Endocrinology/Genetics, Shanghai Institute for Pediatric Research, Xinhua Hospital, School of Medicine, Shanghai Jiao Tong University, Shanghai, China; ^2^ Department of Endocrinology and Metabolism, Henan Key Laboratory of Children’s Genetics and Metabolic Diseases, Children’s Hospital Affiliated to Zhengzhou University, Henan Children’s Hospital, Zhengzhou Children’s Hospital, Zhengzhou, China; ^3^ Center of Neonatal Disease Screening, Jining Maternal and Child Health Care Hospital, Jining, China; ^4^ Center of Neonatal Disease Screening, Jinan Maternal and Child Health Care Hospital, Jinan, China; ^5^ Center of Neonatal Disease Screening, Shijiazhuang Maternal and Child Health Care Hospital, Shijiazhuang, China; ^6^ Center of Neonatal Disease Screening, Shandong Maternal and Child Health Care Hospital, Jinan, China; ^7^ Center of Neonatal Disease Screening, Hefei Maternal and Child Health Care Hospital, Hefei, China

**Keywords:** methylmalonic acidemia combined with homocysteinemia, long-term outcome, newborn screening, MMACHC gene, tandem mass spectrometry

## Abstract

**Objective:** The cblC type of combined methylmalonic acidemia and homocystinuria, an inherited disorder with variable phenotypes, is included in newborn screening (NBS) programs at multiple newborn screening centers in China. The present study aimed to investigate the long-term clinical benefits of screening individual.

**Methods:** A national, retrospective multi-center study of infants with confirmed cblC defect identified by NBS between 2004 and 2020 was conducted. We collected a large cohort of 538 patients and investigated their clinical data in detail, including disease onset, biochemical metabolites, and gene variation, and explored different factors on the prognosis.

**Results:** The long-term outcomes of all patients were evaluated, representing 44.6% for poor outcomes. In our comparison of patients with already occurring clinical signs before treatment to asymptomatic ones, the incidence of intellectual impairment, movement disorders, ocular complications, hydrocephalus, and death were significantly different (*p* < 0.01). The presence of disease onset [Odd ratio (OR) 12.39, 95% CI 5.15–29.81; *p* = 0.000], variants of c.609G>A (OR 2.55, 95% CI 1.49–4.35; *p* = 0.001), and c.567dupT (OR 2.28, 95% CI 1.03–5.05; *p* = 0.042) were independently associated with poor outcomes, especially for neurodevelopmental deterioration.

**Conclusion:** NBS, avoiding major disease-related events and allowing an earlier treatment initiation, appeared to have protective effects on the prognosis of infants with cblC defect.

## 1 Introduction

Methylmalonic acidemia combined with homocysteinemia (OMIM 277400) is a series of inherited autosomal recessive defects in cobalamin [vitamin B12 (VitB12)] metabolism resulting in impaired synthesis of adenosylcobalamin (AdoCbl) and methylcobalamin (MeCbl), which both are essential coenzymes for the conversion of methylmalonyl-CoA to succinyl-CoA (catalyzed by methylmalonyl-CoA mutase) and remethylation of homocysteine to methionine (catalyzed by methionine synthase) (Dionisi-Vici et al., 2006; Lerner-Ellis et al., 2009; Watkins and Rosenblatt, 2011). The cblC type of combined methylmalonic acidemia and homocystinuria is the most common defect in the intracellular cobalamin metabolism pathway (Nogueira et al., 2008), characterized by variable and non-specific symptoms, especially in childhood. The phenotype has a wide expression, with early-onset (before 1 year from birth) often associated with multisystem involvement, neurological deterioration, maculopathy, failure to thrive, cytopenias, renal and hepatic dysfunction (Rosenblatt et al., 1997; Carrillo-Carrasco et al., 2012; Fischer et al., 2014; He et al., 2020). Neurological damage is often with hydrocephalus, cerebral atrophy, white matter abnormalities, and basal ganglia lesions (Weisfeld-Adams et al., 2013; Fischer et al., 2014; Zhang et al., 2019). Developmental delay and intellectual deficits are also frequently impaired (Weisfeld-Adams et al., 2013).

The incidence of cblC defect ranges from 1:46,000 to 1:200,000 in European and American countries (Morel et al., 2006; Weisfeld-Adams et al., 2010) and varies hugely from 1:3,220 to 1:21,488 in China (Zhou et al., 2018; Wang et al., 2019). Previously, individuals with cblC defects were diagnosed symptomatically. However, since tandem mass spectrometry (MS/MS) has been increasingly used in newborn screening (NBS) programs in China, technically enabling identification of more than 30 inherited metabolic diseases (IMDs) by using a single method, it is recommended to identify those infants who are at risk for IMDs, including cblC defect. Most NBS pilot studies have demonstrated a positive effect on early identification of cblC defect by NBS and the neonatal start of treatment on the neurological outcome to a variable extent (Martínez-Morillo et al., 2016; Mütze et al., 2020). However, the impact on the long-term outcome of individuals with cblC defects in the era of newborn screening is yet to be comprehensively explored. This national multicenter retrospective study, therefore, aims at elucidating the long-term outcome of screened individuals and at investigating the impact of different factors on the disease outcome.

## 2 Patients and Methods

### 2.1 Patients

From January 2004 to December 2020, a total of 560 patients with cblC defects identified by NBS were diagnosed and treated at multiple hospitals in China. Among them, 9 cases died and 22 cases were lost to follow-up. All patients were ascertained on DNA-based and MS/MS NBS and most receive personalized treatment as well as regular follow-up. To evaluate clinical outcomes, patients were divided into two groups, with the normal outcome group being healthy and the poor outcome group suffering from varying degrees of physical and mental damage. Among poor outcomes, five different types of clinical parameters were included—intellectual impairment, movement disorders, ocular complications, hydrocephalus, and death. “Intellectual impairment” was categorized based on the presence of developmental delay according to age, or neuropsychological test results, or in the absence of neuropsychological test results on educational level or professional employment. “Movement disorders” mainly manifested as involuntary tremor, gait instability, and disability to walk that developed throughout the disease course, objectified during follow-up examinations by pediatricians and physiotherapists shortly after the symptomatic phase. “Ocular complications” included the presence of strabismus, nystagmus, and a range of ocular findings from unremarkable and mild phenotypes to significant retinopathy. “Hydrocephalus” was detected at cranial MRI, which usually depicted ventriculomegaly, extensive cerebral parenchyma atrophy, and subdural effusion. And the most severe clinical outcome is failure to thrive. Parents or legal guardians of the participants signed an informed consent form, approving analysis of their clinical records, and publication of the anonymous data, in agreement with institutional and national legislation. This study was approved by the Ethics Committee of Xinhua Hospital (approval number XHEC-D-2021-140).

### 2.2 Newborn Screening and Confirmatory Testing

NBS samples of dried blood spots are taken between the ages of 72–120 h according to the recommended time frame (Mak et al., 2013). Blood levels of acylcarnitines, including propionylcarnitine (C3) and acetylcarnitine (C2) were detected by MS/MS (API 4000, American Bio-System Inc., Foster City, CA, United States,) using blood filter papers. The ratio of C3/C2 was calculated at the same time. Second blood spots were requested upon abnormal first-screening results (Niu et al., 2010). To further confirm the diagnosis, urinary organic acids, including methylmalonic acid (MMA) and methylcitric acid (MCA) were measured by gas chromatography-mass spectrometry (GC-MS) (Shimadzu Limited., Shimadzu, Kyoto, Japan, QP 2010) (Al Dhahouri et al., 2018; Al-Dirbashi et al., 2019). Moreover, plasma total homocysteine (tHcy) were assessed using fluorescence polarization immunoassay.

### 2.3 Molecular Genetic Analysis

Genomic DNA was extracted from peripheral blood using the DNA extraction kit (TIANGEN Biotech, Beijing, China) according to the manufacturer’s instructions. All coding exons and flanking regions of *MMACHC* were amplified using polymerase chain reaction (PCR) with primers designed by Primer3 Input (v. 0.4.0, http://bioinfo.ut.ee/primer3-0.4.0/). After PCR, Nucleotide variations were identified using a reference sequence from Genbank (MMACHC: NM_015506). We used the ClinVar database, the HGMD database, and previous literature to identify whether the mutations had been reported. The pathogenicity of novel variants was evaluated based on the American College of Medical Genetics and Genomics (ACMG) standards and guidelines.

### 2.4 Treatment

The treatment of cblC defect varies with different vitamin B12 responsiveness and is adjusted depending on the condition of individual patients (Baumgartner et al., 2014). According to the guideline, the long-term therapy involved parenteral cobalamin, L-carnitine, and oral betaine (Forny et al., 2021). Then, the treatment is adjusted based on levels of biochemical metabolites. Generally, for most patients, intramuscular injections of hydroxocobalamin were the first choice at a dose of 1–20 mg each time, once every 1–20 days, L-carnitine at 50–100 mg/kg/day and oral betaine at 50–100 mg/kg/day.

All patients followed a normal diet. In patients with methionine deficiency, methionine was supplemented orally (Manoli et al., 2016; Huemer et al., 2017).

### 2.5 Statistical Analysis

All statistical analyses were performed using SPSS 24.0 (IBM, Chicago, IL). Continuous data are expressed either as medians and interquartile range or mean ± standard deviation (SD). Categorical variables are presented as percentages (%). The demographic, clinical, and biochemical characteristics were compared between patients with or without symptoms and patients with different prognoses using 2-sample *t* test, χ^2^/Fisher exact test, or Wilcoxon rank-sum test as appropriate. Overall univariate odds ratios and by different categories of poor outcome were calculated using the logistic regression. Multivariate logistic regression analysis was performed using variables that were significantly associated with a poor outcome in univariate analysis to determine the independent risk factors. A value of *p* < 0.05 was considered to indicate statistical significance.

## 3 Results

### 3.1 Study Population

Among all 538 (280 males, 253 females) patients, who were enrolled in our study, 298 (55.4%) were with normal physical and neurocognitive development, 240 (44.6%) had poor outcomes, in which 9 children died of frequent and severe relapses of metabolic decompensations or hydrocephalus. For nearly all patients, data were available from NBS to the last moment of follow-up. Table 1 is a comparison of demographic variables between those with and without poor outcomes. The median age during the follow-up period in two groups was 3.34 years (range 0.63–4.52 years) and 3.8 years (range 0.48–5.25 years), respectively. The age at diagnosis was 1 month (range 0.8–1.5 months) in the normal outcome group, while 1.5 months (range 1–2.2 months) in the poor outcome group. Although all patients were identified by NBS, a total of 201 (37.4%) patients already developed symptoms before treatment, which included poor feeding, vomiting, drowsiness, seizures, developmental delay, and so on. The incidence of variable clinical features was compared between individuals with or without normal physical and neurocognitive development. As shown in Table 1, the incidence of different kinds of initial symptoms was significantly higher in the poor outcome group (*p* < 0.05), which indicates the onset of symptoms might have an adverse impact on the long-term outcome.

**TABLE 1:** comparison of baseline demographic, clinical, and biochemical characteristics between patients with normal and poor outcomes.

Variable	Normal outcome	Poor outcome	*p* value
No.	298 (55.4%)	240 (44.6%)	
Age at diagnosis (months)	1 (0.8–1.5)	1.5 (1–2.2)	
Age during follow-up (years)	3.34 (0.63–4.52)	3.8 (0.48–5.25)	
Biochemical features at NBS			
C3	7.35 (4–22.47)	7.71 (4–31.5)	0.119
C3/C2	0.52 (0.2–9.19)	0.62 (0.21–3.27)	0.000
MMA	78.82 (5–3,253.72)	112.6 (5.3–2,348.8)	0.01
MCA	2.51 (0–397.13)	3.86 (0.17–209.1)	0.000
tHcy	102.99 (31–1,139)	131.2 (31–750)	0.02
Onset of symptoms, n (%)	63 (21.1%)	187 (77.9%)	0.000
Initial symptoms			
Milk refusal, n (%)	37 (12.4%)	114 (47.5%)	0.000
Vomiting, n (%)	29 (9.7%)	71 (29.6%)	0.000
Drowsiness, n (%)	38 (12.8%)	105 (43.8%)	0.000
Seizures, n (%)	13 (4.4%)	41 (17.1%)	0.000
Coma, n (%)	5 (1.7%)	17 (7.1%)	0.002
Dyskinesia, n (%)	8 (2.7%)	82 (34.2%)	0.000
Developmental delay, n (%)	12 (4%)	80 (33.3%)	0.000
Treatment			
With VB12 before onset	248 (83.2%)	89 (37.1%)	
With VB12 after onset	50 (16.8%)	151 (62.9%)	0.000
Biochemical features during follow-up			
C3	3 (0.06–10.83)	3.39 (0.16–13.96)	0.002
C3/C2	0.12 (0.01–0.36)	0.15 (0.02–0.39)	0.001
MMA	4.29 (0–45.19)	6.85 (0–49.57)	0.002
MCA	0.48 (0–4.99)	0.71 (0–4.95)	0.021
tHcy	30.15 (2–59)	35.35 (6.2–59.5)	0.000
Nucleotide variant			
c.609G>A	173 (58.1%)	178 (74.2%)	0.000
c.658_660delAAG	52 (17.4%)	41 (17.1%)	0.911
c.80A>G	55 (18.5%)	26 (10.8%)	0.014
c.567dupT	20 (6.7%)	30 (12.5%)	0.022
c.482G>A	77 (25.8%)	10 (4.2%)	0.000
Others	140 (47%)	134 (55.8%)	0.041

Reference range: C3 in the blood: 0.5–4 μmol/L; C3/C2 in the blood: 0–0.2 μmol/L; MMA in the urine: 0–4 mmol/mol creatinine; MCA in the urine: 0–0.8 mmol/mol creatinine; tHcy in the blood: 0–15 μmol/L.

### 3.2 Biochemical Features

In the study, all the patients were screened by MS/MS presented with elevated C3 levels (>4 μmol/L), an increased C3/C2 ratio (>0.2 μmol/L) in dried blood spots. Subsequently, the increased MMA and MCA levels in urine as well as elevated total plasma homocysteine were exhibited in confirmatory testing. Importantly, the ratio of blood C3/C2, the level of urinary MMA, and MCA in the NBS sample were higher in the poor outcome group than in the normal group (all *p* < 0.05), except for the blood C3 (*p* = 0.119). Data about tHcy levels showed similar differences between the two groups (*p* < 0.05). As can be seen from Table 1, the biochemical markers after treatment showed the difference between individuals with different outcomes, which the blood C3, blood C3/C2 ratio, urinary MMA, urinary MCA, and tHcy were lower in the normal outcome group than those in poor outcome group (all *p* < 0.05). Additionally, all of the biochemical parameters reached the normal range after treatment, laboratory values of blood C3, blood C3/C2 ratio, urinary MMA, urinary MCA, and tHcy showed remarkably decrease tendency than those before treatment, with a significant statistical difference (all *p* < 0.01). As is evident from Table 2, the greatest differences in biochemical data including the blood C3/C2 ratio, urinary MMA, and MGA, tHcy, were observed between asymptomatic or symptomatic subjects as well. However, there were no statistically significant differences in blood C3 at NBS (*p* = 0.087). The urinary MCA at the last available visit between affected infants with or without symptomatic presentation also did not reveal statistically significant results (*p* = 0.086), which may be the reason for there being limited and insufficient data, partly caused by the patient referral process.

**TABLE 2:** Comparison of baseline demographic, clinical, and biochemical characteristics between patients with or without symptoms before treatment.

	Asymptomatic before treatment	Symptomatic before treatment	*p* value
No.	337 (62.6%)	201 (37.4%)	
Age during follow-up (yeas)	3.59 (0.48–4.86)	3.52 (0.67–5.25)	
Age at diagnosis (months)	0.8 (1–1.73)	1.5 (1–2.2)	
Biochemical features at NBS			
C3	7.33 (4–26.13)	7.97 (4.01–31.5)	0.087
C3/C2	0.52 (0.2–9.19)	0.65 (0.22–3.34)	0.000
MMA	85.38 (5–3,253.72)	116.31 (5.3–1,693)	0.022
MCA	2.71 (0–397.13)	3.45 (0.17–209.1)	0.01
tHcy	100 (31–1,139)	134.2 (31–750)	0.001
Biochemical features after treatment			
C3	3.08 (0.26–10.96)	3.66 (0.06–13.96)	0.002
C3/C2	0.12 (0.01–0.37)	0.14 (0.03–0.39)	0.005
MMA	4.7 (0–49.57)	6.45 (0–44.61)	0.036
MCA	0.53 (0–4.99)	0.7 (0–4.95)	0.086
tHcy	30.5 (2–59.5)	35.4 (6.47–59)	0.001
Nucleotide variant			
c.609G>A	205 (60.8%)	146 (72.6%)	0.005
c.658_660delAAG	61 (18.1%)	32 (15.9%)	0.518
c.80A>G	60 (17.8%)	21 (10.4%)	0.021
c.567dupT	24 (7.1%)	26 (12.9%)	0.025
c.482G>A	80 (23.7%)	7 (3.5%)	0.000
others	166 (49.3%)	108 (53.7%)	0.315
Outcome			
Normal	248 (73.6%)	50 (24.9%)	
Poor	89 (26.4%)	151 (75.1%)	0.000
Intellectual impairment	88 (26.1%)	146 (72.6%)	0.000
Movement disorders	39 (11.6%)	65 (32.3%)	0.000
Ocular Complications	6 (1.8%)	25 (12.4%)	0.000
Hydrocephalus	2 (0.6%)	7 (3.5%)	0.016
Deceased	2 (0.6%)	7 (3.5%)	0.016

Reference range: C3 in the blood: 0.5–4 μmol/L; C3/C2 in the blood: 0–0.2 μmol/L; MMA in the urine: 0–4 mmol/mol creatinine; MCA in the urine: 0–0.8 mmol/mol creatinine; tHcy in the blood: 0–15 μmol/L.

### 3.3 Gene Analysis

*MMACHC* mutations were found on both alleles in 510 of 534 patients (95.5%), whereas a single mutation on one allele was noted in 24 patients (4.5%). Among them, 74 different mutations were identified, including 16 nonsense mutations that account for 52.5% (552/1,052) of alleles; the remainder consisted of 18 missense mutations, 3 duplications, 27 deletions, 5 insertions, 5 splice-site mutations. Sequencing analysis identified *MMACHC* mutation in 97.8% (1,052 of 1,076) of all disease alleles. Among them, the common mutation was c.609G>A (p.W203X), c.658_660delAAG (p.K220del), c.482G>A (p.R161Q), c.80A>G (p.Q27R), c.567dupT (p.I190Yfs*13), which accounted for 41.3% (434 of 1,052), 9.2% (97 of 1,052), 9.0% (95 0f 1,052), 8.3% (87 of 1,052) and 4.9% (51 of 1,052), respectively. Between groups with good and poor outcomes or groups whether showed symptoms when therapy was initiated, the types of mutation sites strongly differed. In poor outcome group, the variant of c.609G>A (p.W203X) was more common (74.2% vs. 58.1%), as was c.567dupT (p.I190Yfs*13) (12.5% vs. 6.7%), while these patients appeared to have less incidence of c.482G>A (p.R161Q) (4.2% vs. 25.8%) and c.80A>G (p.Q27R) (10.8% vs. 18.5%) in *MMACHC*. In analogy, c.609G>A (p.W203X) and c.567dupT (p.I190Yfs*13) in *MMACHC* were increasingly observed in symptomatic patients, while c.482G>A (p.R161Q) and c.80A>G (p.Q27R) were more often in asymptomatic patients. Therefore receiving a disease-specific therapy, individuals with these variants in our cohort were more likely to have a better prognosis.

### 3.4 Follow-Up and Outcomes

We evaluated the current health condition of the patients up to December 2020. The prognosis is shown in detail in Table 1. However, of the 240 patients who showed poor prognosis, 234 (97.5%) developed progressive intellectual impairment, 104 (43.3%) manifested movement disorders, 31 (12.9%) exhibited ocular problems, 9 (3.8%) had hydrocephalus and 9 (3.8%) died from disease onset. By dividing them into two groups which depend on whether they suffered from early symptoms of disease before therapy, there is a significant difference between good or poor outcomes (*p* < 0.01). Unfortunately, patients detected by newborn screening but treated after disease onset were more likely to show poor outcomes. In comparison with individuals who remained asymptomatic before treatment, the proportion of intellectual impairment (72.6% vs. 26.1%), movement disorders (32.3% vs. 11.6%), ocular complications (12.4% vs. 1.8%), hydrocephalus (3.5% vs. 0.6%), and death (3.5% vs. 0.6%) were higher in symptomatic group. It is worth noting that among patients deceased, all developed severe symptoms, except two cases without specific symptoms at first who then progressively developed clinical manifestations later even if they received therapy. This might be due to poor treatment compliance.

### 3.5 Factors Affecting Variable Outcomes

Two hundred and forty patients with different types of poor outcomes were analyzed by both univariate and multivariate logistic regression. Factors including the onset of symptoms, nucleotide variants, biochemical markers at NBS, time from onset to treatment initiation were analyzed.

Univariate analysis showed onset of symptom [Odd ratio (OR) 13.16, 95% CI 8.71–19.89; *p* = 0.000], presence of c.609G>A (OR 2.07, 95% CI 1.43–3.00; *p* = 0.000), and c.567dupT in the *MMACHC* gene (OR 1.99, 95% CI 1.1–3.6; *p* = 0.023), baseline C3 (OR 1.06, 95% CI 1.01–1.12; *p* = 0.031) and C3/C2 (OR 1.5, 95% CI 1.04–2.17; *p* = 0.031) were associated with poor outcomes in the whole sample (Table 3). In multivariate logistic regression analysis, onset of symptom (OR 12.39, 95% CI 5.15–29.81; *p* = 0.000), the presence of c.609G>A (OR 2.55, 95% CI 1.49–4.35; *p* = 0.001), and c.567dupT in the *MMACHC* gene (OR 2.28, 95% CI 1.03–5.05; *p* = 0.042) remained independently associated with poor outcomes.

**TABLE 3:** Univariate and multivariate analysis of predictors for poor outcome.

	Univariable analysis			Multivariable analysis		
	OR	95% CI	*p* value	OR	95% CI	*p* value
Onset of symptoms	13.16	8.71–19.89	0.000	12.39	5.15–29.81	0.000
Nucleotide variant						
c.609G>A	2.07	1.43–3.00	0.000	2.55	1.42–3.8	0.001
c.658_660delAAG	0.98	0.62–1.53	0.911			
c.80A>G	0.54	0.33–0.89	0.015			
c.567dupT	1.99	1.1–3.6	0.023	2.28	1.03–5.05	0.042
c.482G>A	0.13	0.06–0.25	0.000			
Others	1.43	1.01–2.01	0.041	1.95	1.23–3.09	0.005
Biochemical features at baseline						
C3	1.06	1.01–1.12	0.031	1.02	0.96–1.08	0.577
C3/C2	1.50	1.04–2.17	0.031	0.78	0.53–1.14	0.194
MMA	1.00	1.00–1.00	0.079			
MCA	1.01	1.00–1.01	0.323			
tHcy	1.00	1.00–1.00	0.057			
With VB12 after onset	8.42	5.63–12.57	0.000	0.82	0.35–2.00	0.66

The results of the logistic regression analysis for the poor outcomes are listed in Table 3. In subjects with intellectual impairment, the multivariate logistic regression analysis showed that onset of symptoms (OR 12.31, 95% CI 5.11–29.66; *p* = 0.000), the mutation of c.609G>A (p.W203X) (OR 2.53, 95% CI 1.48–4.31; *p* = 0.001) and c.567dupT in the *MMACHC* gene (OR 2.57, 95% CI 1.17–5.66; *p* = 0.019) independently predicted the outcome of intellectual impairment (Table 4). While in patients who had ocular abnormalities, the mutation of c.567dupT in the *MMACHC* gene (OR 3.72, 95% CI 1.55–8.94; *p* = 0.003) was an independent predictor as well as the onset of symptoms (OR 5.84, 95% CI 1.13–30.12; *p* = 0.035) (Table 5). In the group with movement disorders, the independent predictor was the onset of disease (OR 13.47, 95% CI 5.6–32.33; *p* = 0.000) as well as the concentration of MMA (OR 1.00, 95% CI 1.00–1.00; *p* = 0.027), whereas the type of nucleotide variant was not significantly correlated with it (Table 6). Additionally, none of the factors were independently associated with the outcome of hydrocephalus (Table 7).

**TABLE 4:** Univariate and multivariate analysis of predictors for intellectual impairment.

	Univariable analysis			Multivariable analysis		
	OR	95% CI	*p* value	OR	95% CI	*p* value
Onset of symptoms	12.15	8.07–18.30	0.000	12.30	5.11–29.66	0.000
Nucleotide variant						
c.609G>A	2.01	1.39–2.91	0.000	2.31	1.41–3.77	0.001
c.658_660delAAG	0.93	0.59–1.46	0.739			
c.80A>G	0.53	0.32–0.88	0.014			
c.567dupT	2.09	1.15–3.78	0.015	2.49	1.17–5.29	0.018
c.482G>A	0.12	0.06–0.24	0.000			
Others	1.52	1.08–2.15	0.016	2.14	1.35–3.39	0.001
Biochemical features at baseline						
C3	1.07	1.02–1.13	0.012	1.03	0.97–1.10	0.287
C3/C2	1.55	1.07–2.25	0.021	0.86	0.61–1.21	0.387
MMA	1.00	1.00–1.00	0.079			
MCA	1.01	1.00–1.01	0.298			
tHcy	1.00	1.00–1.00	0.054			
With VB12 after onset	7.51	5.07–11.14	0.000	0.72	0.31–1.72	0.464

**TABLE 5:** Univariate and multivariate analysis of predictors for ocular complication.

	Univariable analysis			Multivariable analysis		
	OR	95% CI	*p* value	OR	95% CI	*p* value
Onset of symptoms	11.98	3.6–39.92	0.000	5.84	1.13–30.12	0.035
Nucleotide variant						
c.609G>A	0.55	0.26–1.13	0.105			
c.658_660delAAG	1.73	0.75–3.99	0.201			
c.80A>G	0.59	0.18–1.99	0.394			
c.567dupT	4.65	2.01–10.76	0.000	3.72	1.55–8.94	0.003
c.482G>A	0	0	0.997			
Others	1.18	0.57–2.45	0.654			
Biochemical features at baseline						
C3	1.01	0.91–1.12	0.858			
C3/C2	0.79	0.34–1.81	0.570			
MMA	1.00	1.00–1.00	0.708			
MCA	1.01	1.00–1.02	0.189			
tHcy	1.00	1.00–1.00	0.363			
With VB12 after onset	7.84	3.16–19.46	0.000	2.12	0.60–7.42	0.242

**TABLE 6:** Univariate and multivariate analysis of predictors for movement disorders.

	Univariable analysis			Multivariable analysis		
	OR	95% CI	*p* value	OR	95% CI	*p* value
Onset of symptoms	7.87	4.57–13.55	0.000	13.47	5.61–32.33	0.000
Nucleotide variant						
c.609G>A	1.57	0.98–2.52	0.063			
c.658_660delAAG	0.51	0.26–0.99	0.047			
c.80A>G	0.77	0.41–1.45	0.418			
c.567dupT	1.72	0.89–3.32	0.107			
c.482G>A	0.12	0.04–0.4	0.000			
Others	1.40	0.91–2.16	0.125			
Biochemical features at baseline						
C3	0.99	0.93–1.06	0.785			
C3/C2	1.29	0.92–1.80	0.140			
MMA	1.00	1.00–1.00	0.012	1.00	1.00–1.00	0.021
MCA	1.01	1.00–1.02	0.080			
tHcy	1.00	1.00–1.00	0.180			
With VB12 after onset	3.65	2.34–5.70	0.000	0.43	0.20–0.92	0.030

**TABLE 7:** Univariate and multivariate analysis of predictors for hydrocephalus.

	Univariable analysis			Multivariable analysis		
	OR	95% CI	*p* value	OR	95% CI	*p* value
Onset of symptoms	9.49	1.18–76.39	0.035	5.98	0.37–97.21	0.209
Nucleotide variant						
c.609G>A	1.07	0.26–4.31	0.928	1.29	0.69–2.42	0.425
c.658_660delAAG	1.38	0.28–6.73	0.694			
c.80A>G	0.71	0.089–5.77	0.75			
c.567dupT	2.86	0.58–14.17	0.197			
c.482G>A	0.64	0.08–5.21	0.68			
Others	0.48	0.12–1.92	0.297			
Biochemical features at baseline						
C3	0.76	0.55–1.05	0.092			
C3/C2	0.37	0.05–2.90	0.344			
MMA	1.00	1.00–1.00	0.499			
MCA	1.00	0.97–1.03	0.935			
tHcy	1.00	0.99–1.01	0.985			
With VB12 after onset	6.04	1.24–29.38	0.026	1.73	0.21–14.41	0.611

## 4 Discussion

Previous NBS programs enormously improved the health outcomes of affected individuals, since early diagnosis is known to have a great benefit for effectively treating some inborn errors of metabolism (Mütze et al., 2020; Lüders et al., 2021). However, many European countries do not generally advocate widespread screening for the disease of propionate metabolism on the basis that long-term outcomes may not be significantly influenced by early detection (Leonard et al., 2003). In the United States, screening is advocated: it has been argued that even in the absence of clear clinical benefit, early diagnosis enables families to receive genetic counseling more promptly and avoids expensive and time-consuming radiologic and biochemical investigations in affected children. Although there are no national detailed expanded NBS standards in China due to regional differences in the application of MS/MS and lack of valid studies, some work has been done on epidemiology, technical aspects, and clinic validity of MS/MS screening in several provinces and cities in China (Zhang et al., 2021), indicated that NBS programs can prevent irreversible neurological damage and death by allowing treatment to be started, ideally, before the onset of symptoms. We conducted a national multicenter study including 538 patients identified by NBS with confirmed cblC defect to investigate their long-term outcome and to identify the major predictors of the disease outcome.

In the present study, we investigated the clinical and biochemical characteristics of patients with different outcomes to discuss the potential risk factor of poor outcomes. As expected, there were significant differences in clinical phenotypes, pathogenic variants, and biochemical features both at NBS and during follow-up. Meanwhile, infants with poor outcomes appeared to take therapeutic measures after disease onset, conversely, the normal group was more likely to take timely treatment once positive NBS results had been reported at birth, underlining the importance of optimal diagnostic process quality and early NBS results. Importantly, cblC defect is with a high frequency of severe and potentially fatal neonatal decompensations often already during the first days of life (Heringer et al., 2016), a delay in treatment might lead to irreversible damage, pointing out emphatically the importance of pre-symptomatic treatment (He et al., 2020). The mortality rate of patients identified by NBS tended to be lower (Dionisi-Vici et al., 2006). The nine deceased patients in our study, though detected by newborn screening, were all treated after disease onset. Among them, eight died because of recurrent metabolic decompensation and only one died of hydrocephalus, suggesting that onset of the disease is negatively correlated with prognosis.

To identify the effect of onset disease on the long-term outcomes of neonatally screened individuals with cblC defect, we analyzed the detailed information between asymptomatic individuals and symptomatic ones before treatment. In the majority of asymptomatic individuals, the long-term outcomes tended to be more favorable. Unfortunately, two of them appeared no clinical symptoms firstly before therapy but died of acute metabolic crisis due to poor treatment compliance. NBS, though not preventing clinical disease course in all screened individuals, is associated with favorable long-term outcomes. With pre-symptomatic treatment, developmental milestones of the majority of patients detected by newborn screening were normal. These cases benefited from early diagnosis, the timely start of treatment, and the prevention of severe complications.

Regarding biochemical data at NBS, patients with poor prognosis had slightly elevated blood C3/C2 ratio, urinary MCA, but obviously increased plasma tHcy and urinary MMA, which are considered to be correlated with severe brain damage (Kruman et al., 2000; Huemer et al., 2017). However, the relevant metabolic indicators still need to be further explored. Besides, there is no significant difference in the value of blood C3 between groups, which is the opposite of the blood C3/C2 ratio, representing a significant difference. This serves to illustrate that the absolute value of C3 is not sensitive enough since it can be affected by body weight, breastfeeding, and so on. Moreover, compared to severely affected individuals who appeared symptoms before treatment, mildly affected patients who remained asymptomatic showed lower blood C3 and blood C3/C2 ratio concentrations. Interestingly, blood C3 of some patients with the milder disease might not reach the cutoff because of low plasma carnitine obscured elevations in C3 (Ahrens-Nicklas et al., 2015), which results in the possibility of missing mild disease with newborn screening and questioning the sensitivity of newborn screening in identifying these infants. As a consequence, some reports suggested that the C3/C2 ratio can be used as a marker for cblC defect due to the fact that this ratio was elevated on the first and second newborn screens whereas C3 was not above the cutoff (Ahrens-Nicklas et al., 2015). Besides C3/C2 ratio, low methionine could be an additional secondary analyte and was demonstrated to be associated with improved specificity and positive predictive value in one study (Weisfeld-Adams et al., 2010). Moreover, there exists a mild difference in the cut-off value nationwide and a high false-positive or false-negative rates of NBS results based on MS/MS, some experts suggested using next-generation sequencing (NGS) as a second-tier diagnostic test for abnormal NBS results (Luo et al., 2018).

It is noteworthy that genotype-phenotype correlations seem to have a huge impact on the onset of this disorder as well as the prognosis (Lerner-Ellis et al., 2009). For example, in comparison with individuals who remained asymptomatic, those who had already shown clinical manifestations before initiation of treatment were more likely to carry c.609G>A as well as c.567dupT in the *MMACHC* gene. Consistent with previous reports (Liu et al., 2010), both pathogenic variants showed early onset of disease, usually during the neonatal period. This could be related to the fact that c.609G>A variant is nonsense and results in a premature termination codon, which is predicted to cause a truncated or absent MMACHC protein. Similarly, c.567dupT in the *MMACHC* gene, a duplication and frameshift mutation, can also have an adverse effect on the function of this protein. Furthermore, patients carrying these homozygous or heterozygous variants are more likely to develop bad outcomes. On the contrary, individuals who remained asymptomatic before initiation of treatment appeared to carry c.482G>A or c.80A>G in the *MMACHC* gene, both missense pathogenic variants, which predicted excellent clinical outcomes. This result was nearly in line with previous reports (Lerner-Ellis et al., 2009; Huemer et al., 2014; Liu et al., 2020), which described that c.482G>A (p.R161Q) and c.80A>G (p.Q27R) variants were known to predict an attenuated or even benign phenotype with late onset. In a more recent study, Almannai et al. showed that patients with c.482G>A pathogenic variant have a milder disease as indicated by later-onset of symptoms (Almannai et al., 2017), thus when detected by NBS, they were still asymptomatic and once treatment was to be started, the irreversible damage caused by the onset of symptoms could be prevented.

Our findings suggested that symptomatic onset, independently from other predictors like pathogenic variant, metabolic analytes, and treatment, is a strong predictor of poor outcomes, such as intellectual impairment, movement disorders, ocular complications except for hydrocephalus. But the underlying mechanism remains obscure. Results from this current study also suggested that c.609G>A and c.567dupT variants were independent factors of poor prognosis, especially for intellectual impairment. In addition, the odds ratio of c.609G>A variant is a little higher than that of c.567dupT variant in poor outcome, but when intellectual impairment was analyzed, the odds ratio of c.567dupT variant was higher than that of c.609G>A variant. Considering a much higher carrier prevalence of c.609G>A in the *MMACHC* gene among the Chinese population, we proposed that c.609G>A mutant allele, the mutational hotspots, could be potential candidates for gene screening and should be listed in the pre-pregnancy carrier screening panel in China (Qiao et al., 2020). Furthermore, eye diseases are common in patients with the *MMACHC* gene c.271dupA homozygous mutation and are characterized by early maculopathy (Gizicki et al., 2014; Brooks et al., 2016). Ruxuan He et al. showed that the incidence of ocular problems in patients with the c.609G>A variant is much lower (He et al., 2020). And in our study, there is a strong link between c.567dupT variant and ocular abnormalities which is not reported before. This may be because we included the presence of strabismus and nystagmus, which are obvious phenotypes, as criteria of ocular complications. Due to the lack of formal assessment of ocular complications, this result remains to be investigated in the future. In addition, our study found that MMA seems to be a strong biochemical marker of movement disorder because MMA is associated with a high risk of basal ganglia stroke (Dionisi-Vici et al., 2006). This result substantiates the fact that movement disorder could be recovered after treatment once MMA decreases (Matos et al., 2013). However, in our study, we did not observe any risk factors that independently predict the outcome of hydrocephalus.

Although the incidence of cblC defect is low, the outcomes of affected patients are not favorable. The rate of death in our cohort (3.8%) is lower than that of a historical cohort diagnosed before initiation of NBS (26%) (Rosenblatt et al., 1997). In addition, compared to previous studies (Liu et al., 2018; He et al., 2020), the proportion of poor outcomes as well as mortality in our study is lower, demonstrating that NBS reduces mortality and allows a favorable outcome. For late-onset patients, there is no doubt that early detection by NBS and timely treatment improved long-term outcomes (Weisfeld-Adams et al., 2010). Several studies have demonstrated that early-onset patients, though identified by NBS, still have suboptimal outcomes with a high residual burden of neurologic and ophthalmologic disease (Weisfeld-Adams et al., 2013; Ahrens-Nicklas et al., 2017). In our study, NBS data were typically reported at 2–3 days of life, which enable early diagnostic treatment, thus reducing the frequency of hospitalization and severity of symptoms. Overall, based on our large sample, NBS for cblC defect should be considered since survival and prevention of severe complications in early-onset patients can be improved by early treatment (Dionisi-Vici et al., 2006; Weisfeld-Adams et al., 2010; Huemer et al., 2015; Huemer et al., 2017).

In all screened individuals, there is still a minority at risk of mild to severe psychomotor impairment or complications. This might be the reason that some patients had already developed neonatal disease manifestations, therefore initiation of treatment with intramuscular hydroxocobalamin should even precede recalling screening.

## 5 Conclusion

The present study has discussed a large cohort of individuals with cblC defects. Our study emphasizes the factors affecting outcomes in individuals with cblC defects. Those with a better prognosis have milder diseases as indicated by less symptomatic manifestations, less prominent biochemical abnormalities on NBS, and easier control as made evident by the biochemical profile on the last follow-up. The onset of symptoms is an independent risk factor for poorer outcomes and independently carries a greater risk than pathogenic variants. Newborn screening facilitates early diagnosis, improves the quality of therapy, allows longer survival, and prevents the damage of symptoms, which is the most important prognostic factor. There is no doubt that NBS is a highly successful preventive care program, resulting in favorable clinical outcomes for screened individuals.

## Data Availability

The original contributions presented in the study are included in the article/supplementary files, further inquiries can be directed to the corresponding author.
